# AllerBase: a comprehensive allergen knowledgebase

**DOI:** 10.1093/database/bax066

**Published:** 2017-09-12

**Authors:** Kiran Kadam, Rajiv Karbhal, V. K. Jayaraman, Sangeeta Sawant, Urmila Kulkarni-Kale

**Affiliations:** 1 Bioinformatics Centre; 2 Centre for Modeling and Simulation, Savitribai Phule Pune University, Pune 411007, India; 3 Shiv Nadar University, Gautam Buddha Nagar, Uttar Pradesh 201314, India

## Abstract

Allergic diseases represent a major health concern worldwide due to steady rise in their prevalence leading to increased disease burden. The field of allergy research has witnessed significant progress and the focus of studies has shifted to molecular level. Vast amounts of data for allergens are archived in allergen databases, which cover diverse aspects of allergens and allergenicity with varying degrees of completeness. Users are required to refer to multiple databases including general purpose immunological databases to obtain relevant data of allergens. AllerBase, a relational database, has been developed with the objective of integrating protein allergens and related data from prevailing bioinformatics resources and published literature. AllerBase is a manually curated comprehensive knowledgebase of experimentally validated allergens where various attributes of allergenicity are made available on a single platform. Links to sequences, structures and immunological data are provided, where available, for all allergens. Data for specific features such as IgE-binding epitopes, IgE cross-reactivity and IgE antibodies are curated and archived. Bibliographic data reporting assays used for experimental characterization of allergens are compiled and processed using text mining approach. AllerBase, thus provides enhanced coverage of data with high granularity. The database can be browsed by category of allergens, epitopes or antibodies. It can also be queried using various attributes such as allergen name, isoallergens/variants, taxonomic level, source organism, sequences, structures, epitopes, antibodies and cross-reactive allergens. AllerBase also provides interface for sequence-based analyses and visualization of structure/epitope which can be used as a framework for translational research. A Completeness Index has been devised to indicate the availability of data on nine attributes for every allergen. This index will also serve as a pointer to identify allergen-specific areas of further research. AllerBase can be used to understand various aspects of allergy and allergenicity at molecular level and to design anti-allergy therapeutics.

**Database URL:** http://bioinfo.net.in/AllerBase/Home.html

## Introduction

Allergic reactions to various substances from diverse sources are one of the major health problems. A significant increase in prevalence of allergic diseases has been reported in industrialized and developed countries in the past few years (1). Allergy or Type I hypersensitivity is the inappropriate immune response in genetically predisposed individuals. This immune response is elicited against a particular type of antigens commonly referred to as allergens, resulting in production of immunoglobulin E (IgE) (2). Majority of allergens are proteins which show significant variability in terms of their abundance and potency. The sources of these allergens include consumed substances (food allergens), inhaled substances (aeroallergens) or venoms which are mainly derived from variety of plants, animals and fungi.

Protein allergens possess certain immunological characteristics which play important roles in their allergenicity. Allergens bind IgE through two or more IgE-binding epitopes or antigenic determinants which in turn facilitates cross-linking of allergens with basophils and mast cells. IgE-binding epitopes can either be linear (sequential) or conformational (discontinuous) in nature and they are critical for elicitation of clinical symptoms of allergy (3, 4). Allergic cross-reactivity is a phenomenon characterized by recognition of multiple allergens by a specific antibody, which provides important insights into allergic diseases and their diagnosis (5, 6). It has been shown that structural similarity of allergens and specifically that of IgE-binding epitopes plays a critical role in cross-reactivity (7, 8).

Over the years, advances in genomic and proteomic techniques have led to significant progress in the field of allergy research. In spite of significant increase in the number of characterized protein allergens and better understanding of their functions, the common biochemical and structural features that define allergenicity of these molecules have not been conclusively found. In the view of challenges like dramatic rise in food allergies and allergy assessment of genetically modified foods, several bioinformatics methods have been developed which deal with assessment of allergenicity and help in better understanding of allergy (9).

The growth in allergen data has given rise to many allergen-specific databases. These databases archive useful data for allergens and differ from each other with respect to the main focus, type of data archived and applications (10). The Allergen Nomenclature Sub-Committee of the World Health Organization (WHO) and International Union of Immunological Societies (IUIS) have developed an unambiguous and systematic nomenclature system for protein allergens (11). This WHO/IUIS Allergen Nomenclature Sub-Committee has developed and maintains the official Allergen database (http://www.allergen.org/). This database also archives isoallergens and variants of allergens and it is updated regularly (12). Although WHO/IUIS Allergen database enlists majority of the characterized allergens, it does not represent the total allergen data as there are large number of allergens reported in literature that are not recognized by WHO/IUIS. Allergome (http://www.allergome.org) on the other hand is a comprehensive database that archives WHO/IUIS approved and other allergens reported in literature (13). The Structural Database of Allergenic Proteins (SDAP, http://fermi.utmb.edu/SDAP) is an allergen database with an emphasis on structural biology of allergens (14). It archives 3D models for allergens for which experimental structures are not available. However, the information regarding structure predictions is rather limited. The Allergen Database for Food Safety (ADFS, http://allergen.nihs.go.jp/ADFS) archives data on protein allergens and small molecule allergenic compounds with considerable overlap with existing resources (15). AllergenOnline (http://www.allergenonline.org) is an important allergen database that documents a list of peer reviewed allergens, which is curated by an expert review panel using stringent criteria (16). It also consists of sequence search routines that can be utilized to compare allergen sequences with user-provided sequences including proteins which are recommended for allergenicity risk assessment before their introduction into genetically modified crops. The AllFam database (http://www.meduniwien.ac.at/allfam/) is an important resource as it deals with classification of allergens into protein families by integrating allergen data from WHO/IUIS Allergen and AllergenOnline databases and protein family data from Pfam (17, 18). The Immune Epitope Database (IEDB, http://www.immuneepitope.org) is an extensive database of experimentally validated epitopes along with corresponding reactivity data. It also archives epitope data associated with allergic reactions (19). The international ImMunoGeneTics database (IMGT, http://www.imgt.org) is a comprehensive and integrated immunological resource, which also stores data for IgE antibodies (20). AgAbDb, which is an antigen-antibody interaction database developed in-house (http://bioinfo.net.in/AgAbDb.htm), also documents molecular data on allergen-antibody interactions derived from co-crystal structures of allergen-antibody complexes (21, 22). Besides the aforementioned databases that specifically store allergenicity-related and immunological data, primary bioinformatics databases such as GenBank (23), GenPept (24), UniProtKB (25) and Protein Data Bank (PDB) (26) also archive data on various aspects of allergens.

The advances in allergy research in recent times have given rise to accumulation of significant amount of data on experimental validation of allergens as well as on molecular features like IgE-binding epitopes, IgE cross-reactivity, IgE antibodies, etc. Thorough study of these attributes is necessary to gain further understanding of allergic reactions, allergens as well as to develop strategies for prevention and treatment of allergies. The primary requirement to undertake such studies will be the timely integration of existing heterogeneous allergen-related data in literature and existing databases with access to it. Although existing allergen databases have been very useful, the data archived in these databases for all of these attributes is either lacking or it is not uniformly archived.

AllerBase, a comprehensive knowledgebase of allergen data has been developed and presented here. The database comprises of experimentally validated allergens which have been compiled by extensive search and manual curation from the existing resources and published literature. AllerBase is the first allergen database that includes both, detailed information about experimental validation of allergens as well as data on allergen-specific features such as IgE-binding epitopes, IgE antibodies and IgE cross-reactivity.

## Materials and methods

### Data compilation and curation

The allergen and allergen-related data were compiled by integration and curation of data from existing resources and published literature. The types of data archived in AllerBase and the resources from which each data type was extracted are shown in Table 1.

**Table 1.** Resources/databases used for compilation and curation of data in AllerBase

Type of data	Resources/databases
Allergens and isoallergens/variants	WHO/IUIS-Allergen, Allergome, AllergenOnline, GenBank, GenPept, UniProtKB, PDB, PubMed
Experimental validation (assay/test with reference/s)	PubMed
Allergen sequences	GenBank, GenPept, UniProtKB, PDB
Allergen 3D structures	PDB
IgE-binding epitopes	IEDB, AgAbDb, IMGT/3Dstructure-DB, PubMed
IgE antibody sequences	IMGT-LIGM, GenPept, PubMed
IgE cross-reactivity	PubMed

#### Allergen data

Data from allergen databases and primary databases were retrieved by using their respective search utilities and appropriate keywords. For extracting allergen and allergen-related data from literature, an extensive literature search in PubMed (27) was performed by employing MeSH terms along with keywords specific for allergens in general and source organisms in particular. The large amount of bibliographic data obtained from the search results were subjected to further curation by using a text mining approach. Perl scripts were written to extract the relevant references using predefined keywords. Data for experimentally validated allergens from shortlisted references were further curated manually.

#### Assays/methods data

Experimental characterization and assessment of allergenicity and immunological features of an allergen using *in vivo* and *in vitro* assays/methods represents an important aspect of allergy research (28–30). Data on experimental validation for each allergen representing physicochemical and immunological assays/methods were compiled along with respective bibliographic references. The text mining approach described earlier was used for data curation.

#### IgE epitope data

IgE epitope data from corresponding databases were retrieved by using their respective search utilities. For instance, epitope data from IEDB (19) was retrieved by using source antigen as the search parameter and by providing a particular ‘Organism’ and/or ‘Antigen Name’ as the search criteria. The epitope data obtained from IEDB, AgAbDb (21, 22) and IMGT/3Dstructure-DB (31) databases as well as additional epitope data extracted from the literature were further curated manually based on corresponding references prior to populating AllerBase.

#### IgE antibody data

IgE antibody data in AllerBase consist of nucleotide and protein sequences of light and heavy chains as well as single chain antibody fragments. IgE sequences from IMGT-LIGM (32) were extracted by querying the database for immunoglobulins with ‘Epsilon’ heavy chain. It must be mentioned that a significant amount of IgE antibody data available in IMGT-LIGM cannot be retrieved in query-enabled form due to lack of explicit annotations for heavy chain type as ‘Epsilon’, though queries based on accession IDs enable retrieval of such antibody data. IgE data from GenPept and published literature were curated by employing keyword-based searches using the terms such as ‘IgE’, ‘antibody’, ‘transcript’, etc.

#### IgE cross-reactivity data

Data for IgE cross-reactivity among the allergens were compiled from the published literature. PubMed was searched for every allergen by combining allergen/protein name with terms such as ‘cross-reactivity’, ‘cross-reactive’, ‘cross-reacting’, etc. The results obtained were manually curated to acquire relevant data on allergen cross-reactivity.

#### Criteria for data inclusion

Experimental validation was considered as the criterion for inclusion of allergens and their features such as IgE epitopes, IgE antibodies and IgE cross-reactivity in AllerBase. In recent years, number of allergens has been characterized by employing the highly efficient proteomics approach which involves identification of novel allergens using analytical techniques like mass spectrometry (33, 34). As a result of these studies, many novel allergens have been identified which do not possess molecular data like sequence or structure even though their identity as allergen has been established. These allergens represent important component of modern allergy research and therefore they are also archived in AllerBase.

**Figure 1.**
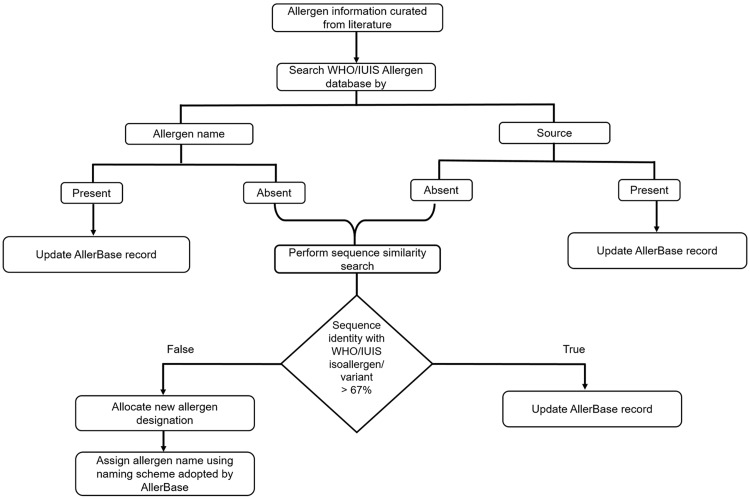
Systematic approach employed for inclusion of curated allergens.

A systematic approach was employed for inclusion of allergens curated from published literature, which are not archived in WHO/IUIS Allergen database. The steps employed in the approach are illustrated in Figure 1. For these allergens, the designation as isoallergens/variants of existing allergens or new allergens was provided using the criterion defined by Allergen Nomenclature Sub-Committee of the WHO/IUIS, which is based on percent sequence identity cut-off of 67%.

#### Allergen naming scheme

Each allergen documented in AllerBase has been provided with a specific name. Allergens approved by WHO/IUIS have been designated with the same name provided by the Allergen Nomenclature Sub-Committee of the WHO and IUIS and are provided with a pointer indicating that they are officially recognized by WHO/IUIS. New allergens that are yet to be designated by WHO/IUIS are named using either of the following strategies. (i) Allergens for which names have been assigned by the authors in the relevant published research article, the same names have been retained so as to maintain the consistency of names in literature and AllerBase. (ii) For naming rest of the allergens, AllerBase adopts a strategy similar to WHO/IUIS nomenclature wherein the first two components of an allergen name are derived from the species and genus name of the allergen source. The last component is derived from the abbreviation of the protein name in place of the number as used in WHO/IUIS-Allergen naming system.

#### Database update

AllerBase will be updated on a weekly basis based on the availability of newly published data on allergens and its attributes.

### Database implementation and web interface

AllerBase is a relational database developed on the MS Windows operating system using MySQL server 5. Apache HTTP Server (Version-2.2) is used as a web server. The query system has been developed using PHP. The web interface is developed using HTML, CSS and JavaScript. Utilities for analysing the allergen nucleotide/protein sequences were also integrated in the database. These include standalone version of NCBI BLAST (version 2.2.29+) for sequence-based database searches (35) and MUSCLE (version 3.8) for multiple sequence alignment (36). Water and Needle programmes available in EMBOSS package at the EMBL-EBI server are also integrated using Perl scripts for local and global pairwise sequence alignments (37–39).

### AllerBase: data organization and salient features

**Figure 2.**
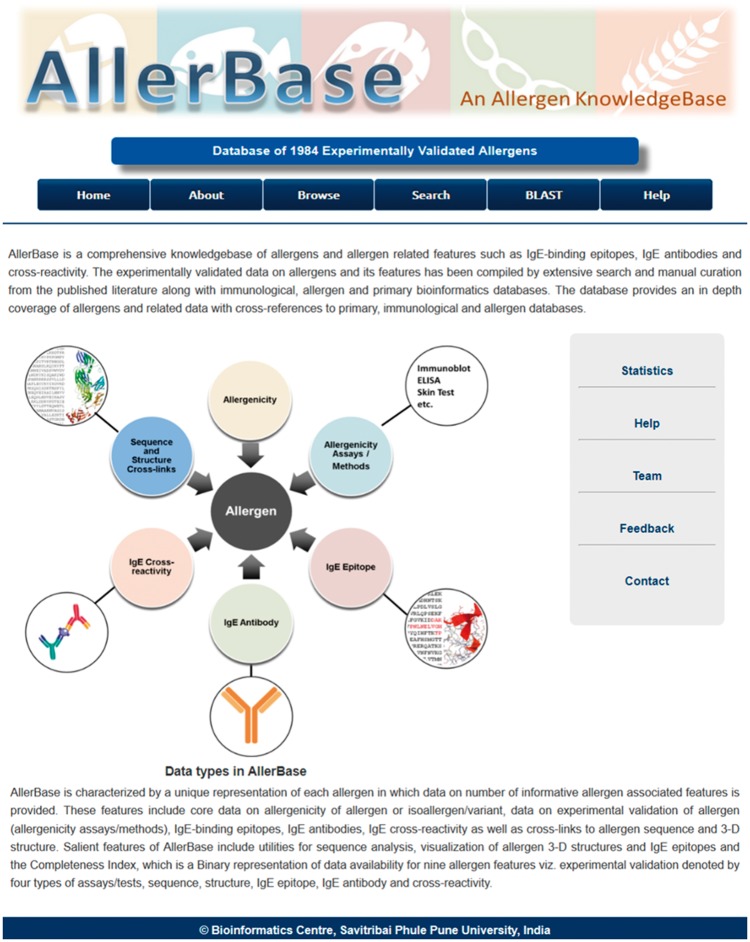
Snapshot of AllerBase home page.

AllerBase is an extensive and curated database of experimentally validated allergens and allergen-related features (Figure 2). Currently, AllerBase documents data on 1984 experimentally validated allergens along with detailed account of allergen-related features. The current statistics of AllerBase are given in Table 2. The database is normalized up to third normal form (3NF) to eliminate data redundancy and anomalies associated with data insertion, update and deletion. AllerBase offers high level of granularity for allergen data as it consists of basic information on allergenicity as well as details of molecular attributes such as sequence, structure and IgE-binding epitopes.

**Table 2.** Current statistics of data in AllerBase^a^

Type of data	Number of entries
Total number of unique allergens	1984
Allergens from plants	889
Allergens from animals	853
Allergens from fungi	221
Allergens from bacteria	20
Allergens from virus	1
Allergens without isoallergens/variants	1117
Number of isoallergens/variants	1271
Total number of allergens and isoallergens/variants	2388
Allergens with 3D structures	251
Allergen-antibody co-crystal structure complexes	10
Allergens with experimentally validated IgE epitopes	143
Number of distinct IgE-binding epitopes	855
Allergens showing IgE cross-reactivity	558
IgE cross-reactivity relations	879
IgE antibody sequences	1877
Allergen specific IgE antibodies	440

a as on 20 August 2017.

**Figure 3.**
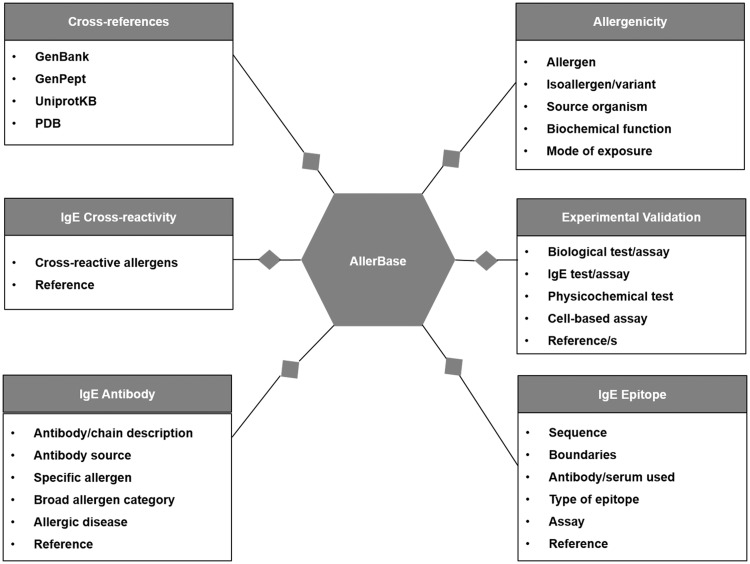
Schematic representation of data types in AllerBase.

AllerBase archives core data associated with allergens, experimental assays (Methods), IgE epitopes, IgE antibodies, IgE cross-reactivity and cross-links to molecular data like sequence and structure. A schematic representation of the database is shown in Figure 3. Cross-references provided to other databases further enhance the utility of allergen data in AllerBase.

A typical allergen entry includes a unique AllerBase ID for each allergen along with core data such as allergen name, source organism, kingdom, biological function (role) and mode of exposure. Complete information on isoallergens/variants of allergens, where available, is also archived. Cross-links to primary databases that archive molecular data are provided for every allergen and/or its isoallergen/variant. These databases include GenBank (23), GenPept (24), UniProtKB (25) and PDB (26). Cross-references to major allergen databases such as IUIS-Allergen (11), Allergome (13) and AllFam (17) are also given as applicable.

**Figure 4.**
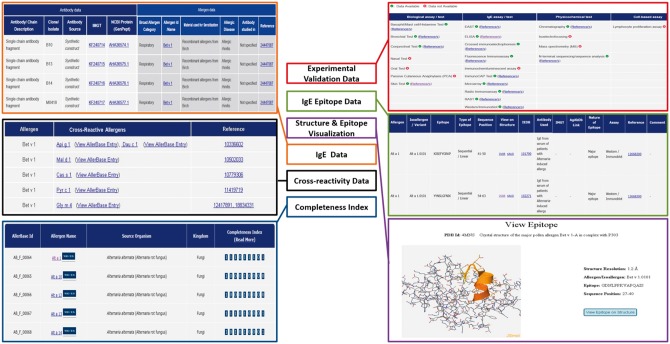
Salient features of AllerBase.

Data for experimental validation of allergens along with the assays/methods used for allergen characterization is also archived in AllerBase. These methods/assays are grouped into four categories. (i) Biological tests/assays used as diagnostic tools include skin test, nasal provocation test, oral test, bronchial test, conjunctival test, passive cutaneous anaphylaxis and Basophil/Mast cell/Histamine-based assay; (ii) IgE tests/assays which comprise of IgE antibody-based assays such as enzyme-allergosorbent test (EAST), crossed immunoelectrophoresis, enzyme-linked immunosorbent assay (ELISA), fluorescence immunoassay, immunochemiluminescent assay, ImmunoCAP, microarray, radioallergosorbent test (RAST), radio immunoassay and western/immunoblotting assay; (iii) physicochemical tests employed for allergen characterization like chromatography, isoelectrofocusing, mass spectrometry and N-terminal sequencing and (iv) cell-based assay like lymphocyte proliferation assay which studies effects of allergen on specific cells. Thus, data for 10 IgE assays/tests, 7 biological assays/tests, 4 physicochemical tests and 1 cell-based assay are documented in AllerBase, as per availability, for each allergen (Figure 4). Bibliographic data for the assays and tests includes references on human-based as well as animal model(s)-based characterization of allergens. Archival of such data is important because animal models are routinely employed for studying allergic responses and they have contributed significantly to the understanding of immunological mechanisms underlying allergic diseases (40, 41). The complete list of references associated with a particular assay/test for each allergen is provided on a separate page. Each reference is represented either by its PubMed ID or the digital object identifier with cross-link to the journal page. For each PubMed ID, details such as journal name, year and volume along with title of the paper and author names are given. The users can select the PubMed ID/s using checkboxes provided and download the abstracts.

IgE epitope data in AllerBase include epitope sequence/s with boundaries, assay/s used for the characterization as well as cross-references to immunological databases such as IEDB (19) and/or IMGT/3Dstructure-DB (31). Additional annotations of epitopes provided in AllerBase include their type viz. sequential or conformational and immunodominant/major/minor or strong/moderate/weakly binding based on their antibody-binding affinity (Figure 4). This information on classification of epitopes is manually curated from the relevant published references and is retained as provided by the authors of respective publications.

One of the important features of AllerBase is archival of IgE antibody data that comprise of nucleotide and protein sequences of IgE including light and heavy chains as well as single chain antibody fragments. Majority of these sequences belong to biological sources like Human, Mouse while some are synthetic constructs. IgE antibodies for which data are archived in AllerBase are generated in response to (i) a specific allergen e.g. Bet v 1 or (ii) allergens belonging to broad categories such as food allergens or (iii) allergic diseases such as atopic dermatitis. Each antibody is provided with detailed annotation which includes basic description of the antibody/chain, source and clone/isolate from which the antibody is produced along with cross-links to IMGT/LIGM-DB (32) and GenPept (24) databases. Allergen associated information like the broad allergen category, specific allergen id/name, material used for sensitization, allergic disease and patient/population in which the antibody has been raised and studied is also provided along with cross-link to the corresponding publication (Figure 4).

A prominent aspect of AllerBase is the availability of cross-reactivity data and interlinking of cross-reactive allergens (Figure 4). This feature facilitates the user to retrieve a list of all cross-reactive allergens by specifying an allergen name. For each allergen in this list, its corresponding cross-reactive allergens can further be retrieved through the cross-links provided. This will enable users to review a network of cross-reactive allergens and compare them using features like sequence similarity and membership to a particular allergen family, if any.

Evolutionary Trace (ET) represents an efficient and widely used method for identification of functionally critical residues in proteins with known structures (42). The method utilizes sequence similarity in a group of homologous proteins to rank amino acids in a protein sequence with respect to their relative evolutionary importance and demonstrate a structural map of these residues. Evolutionary trace report maker assembles information about protein sequence, structure and annotations from different sources and implements real-valued ET (43). It accepts PDB ID or UniProtKB accession number as input and provides a human-readable document and other supplementary files of the analysis as an output. For each allergen structure in AllerBase (PDB ID), a cross-link to download pre-computed ET report file is provided.

### User interface

AllerBase provides a simple and user-friendly web interface for efficient searching, browsing and visualization of allergen and related data.

### Browse utility

The ‘Browse’ utility of AllerBase provides the user with an option to access allergen, IgE epitope and IgE antibody data in a single step. The ‘browse allergens’ option is provided for browsing the allergen data by selecting taxonomic levels such as animals, plants, fungi, bacteria and viruses. The ‘browse IgE epitope’ option is provided for retrieving sequential (linear) and conformational (discontinuous) epitope data. ‘IgE antibodies’ can be browsed by selecting one of the four options, viz. antibodies associated with (i) specific allergen, (ii) broad allergen category (e.g. respiratory, food and contact), (iii) allergic diseases (e.g. allergic asthma, allergic rhinitis and atopic dermatitis) and (iv) none of the specific allergens and/or allergic diseases. 

### Search utility

The search utilities in AllerBase are of two types; viz. Basic Search and Advanced Search.

#### Basic search

**Figure 5.**
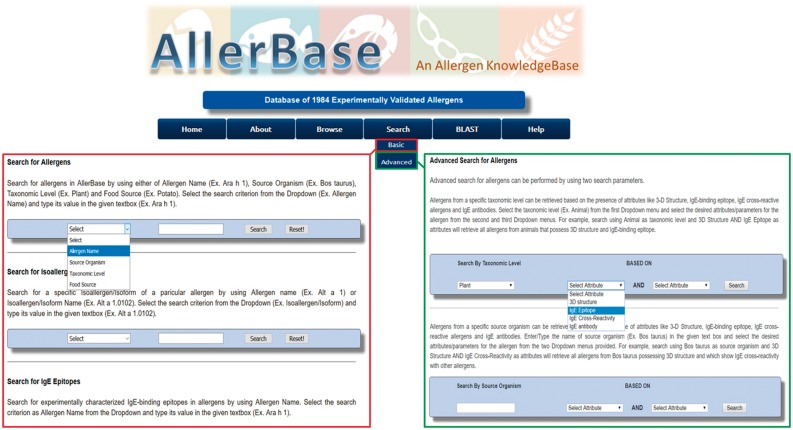
Basic and advanced search utilities of AllerBase.

The Basic Search utilities of AllerBase facilitate search and retrieval of varied data types such as allergens, isoallergens/variants, epitopes, IgE antibodies and cross-reactive allergens (Figure 5). For example, users can search for allergens using allergen name, source organism, taxonomic level or food source. Isoallergens/variants of an allergen can also be searched by providing the allergen name or isoallergen name. Basic search can also be utilized to search for IgE epitopes and cross-reactive allergens using allergen name as a search parameter. Search for IgE antibody data can be performed via basic search by specifying allergen name, broad allergen category, allergic disease, antibody source organism or PubMed ID of the reference study.

#### Advanced search

Advanced search facility of AllerBase is an exhaustive search utility. It facilitates the user to formulate specific queries based on a combination of up to three features of allergen/s. The users can search the database and retrieve allergens belonging to a particular taxonomic level or source organism, combined with the criterion of availability of any one or two out of four allergen features (attributes) viz. 3D structure, IgE epitope, IgE antibody and IgE cross-reactivity (Figure 5). Advanced search utility can also be used to search AllerBase by the type of IgE epitope (sequential/conformational) in combination with allergen name or source organism. Similarly, a utility to search IgE epitopes according to binding affinity (immunodominant/major/minor/strong/moderate/weakly binding) in combination with name of allergen or source organism is also provided, which is a unique feature of advanced search utility of AllerBase.

AllerBase also provides the option to download and save the data retrieved in response to various queries in text format.

#### Sequence analysis

To enable basic analysis of nucleotide and/or protein sequences of an allergen, sequence analysis utilities are provided in AllerBase. A user can perform BLAST search using a nucleotide/protein sequence of an allergen/isoallergen/variant as a query against all the nucleotide/protein sequences archived in AllerBase (35). A user can also search nucleotide or protein sequence of novel allergen by pasting the same in the given textbox or uploading it from a saved file to carry out BLAST search against AllerBase. The BLAST search can be performed using either the default *E*-value cut-off of 0.01 or a user defined *E*-value. Further, multiple sequence alignment of the hits obtained from BLAST searches can be performed and resulting alignment files can be downloaded (36). A utility to perform pairwise sequence alignment of nucleotide and/or protein sequence of an allergen archived in AllerBase and a user-specified sequence has also been provided (37–39).

#### Structure and epitope visualization

A structure visualization utility based on JSmol along with its corresponding functionalities has been integrated in AllerBase to visualize complete 3D structures of allergens and/or allergen-antibody co-crystal structures (where available). The list of PDB ID codes of 3D structures is provided with each PDB ID linked to the corresponding 3D structure. This utility also enables visualization of both sequential and conformational epitopes which will aid in gaining insights into characteristics of IgE epitopes as well as allergen-antibody interactions (Figure 4).

#### Completeness Index

AllerBase provides a Completeness Index for each allergen, which represents availability of data for nine specific features (attributes) of allergens in a fixed order. These features include biological assay/test, IgE assay/test, physicochemical test, cell-based assay, sequence, structure, epitope, IgE antibody and cross-reactivity. It has been observed that the availability of data for these attributes of allergens varies and hence it becomes relevant for a user to know extent of available data in one glance. The Completeness Index is a binary representation in which the indices denoting allergen attributes are pre-defined and where ‘1’ denotes availability of data for a particular attribute while ‘0’ denotes non-availability. The Completeness Index is an integral part of every allergen entry and is also displayed with respective allergen as an outcome of search results for allergens (Figure 4).

## Discussion

Integration of allergy-related data from various resources is very important for their efficient and comprehensive utilization for knowledge generation and applications. Heterogeneity in data sources as well as in organization of data in databases presents a major limitation for integration of data (44). AllerBase represents an important step towards easy access to diverse yet integrated allergen data for an in-depth understanding and analysis of allergens. It integrates different types of data associated with allergens from several resources as well as provides cross-references to large number of experimental studies.

The extent of experimental evidence of allergenicity varies across various allergen databases such as Allergome, WHO/IUIS and SDAP. Archival of extensive data on experimental validation of each allergen is a common feature of both, AllerBase and Allergome databases. In addition to bibliographic data for studies on allergenicity, Allergome also archives references for studies on animal models, diagnosis, epidemiology, molecular biology, etc. However, it also documents a number of proteins that are not proven allergens and therefore user needs to process Allergome data to filter non-allergen entries, if any. AllerBase, on the other hand, stores only well-curated bibliographic data which strictly pertains to validation of allergenicity and thus provides only the related references thereby maintaining accuracy and granularity of the data.

IgE epitope data archived in AllerBase is extensively annotated along with cross-links to important immunological databases. For instance, a discontinuous epitope belonging to cockroach allergen Bla g 2 is provided with cross-links to IEDB (19), IMGT/3Dstructure-DB (31) and AgAbDb (21, 22) databases, each of which archives various types of data associated with this epitope which ranges from amino acid sequence to molecular interaction with respective antibody. This represents a unique feature of data integration from multiple resources, which has not been included in any of the allergen databases that are currently available in the public domain.

IgE antibody data in terms of sequences and/or structures are available in primary databases; however, the derived data associating IgE with respective allergen (and/or allergic condition) is not available, especially in query-enabled form. The archival of IgE sequences associated with particular allergens and/or allergic diseases in AllerBase can be of immense value from the point of view of studying IgE repertoire associated with allergic conditions. IgE cross-reactivity data mined from the literature is also incorporated in AllerBase. Such data on cross-reactivity of allergens is provided in Allergome but it needs further processing by the user as the experimental evidence of cross-reactivity is not explicitly provided. On the other hand, AllerBase provides a list of cross-reactive allergens for a given allergen along with the reference/s to the experimental studies.

**Figure 6.**
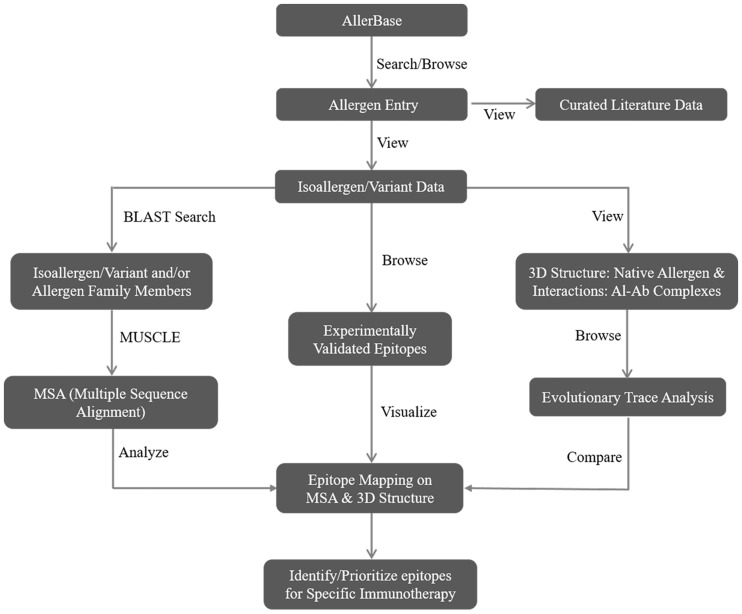
A sample workflow representing application of AllerBase.

Data archived in AllerBase will be useful for development of computational algorithms for prediction of allergens/allergenicity, IgE-binding epitopes and allergenic cross-reactivity. Figure 6 illustrates a workflow demonstrating utility of AllerBase towards designing specific immunotherapy.

Observations from the study of allergy-related literature indicate that there are still important issues which need to be addressed. For instance, the molecular details available for a particular allergen and data obtained from epidemiological studies for specific population as well as clinical records of patients are often archived in independent resources. Integration of such kind of data represents a significant challenge.

## Conclusions

AllerBase, a comprehensive allergen knowledgebase, has been developed that comprises of curated data on experimentally validated allergens and important allergen features such as allergenicity, experimental validation, IgE epitopes, IgE cross-reactivity and IgE antibodies. AllerBase is characterized by extensive integration of data on allergens, IgE antibodies and the development of Completeness Index. For every allergen, this index can be used to summarize extent of available data and to indicate scope for further research, as applicable. Availability of the data and tools for sequence and structure analysis makes AllerBase a platform for design and development of anti-allergy therapeutics.
